# Efficacy and Safety of a Formulated Herbal Granula, Jiu Wei Zhen Xin, for Generalized Anxiety Disorder: A Meta-Analysis

**DOI:** 10.1155/2018/9090181

**Published:** 2018-03-07

**Authors:** Sheng Wang, Lin-lin Zhao, Xin-jian Qiu, Dong-sheng Wang, Tao Tang, Jie-kun Luo, Sui-yu Hu, Hui-yong Huang

**Affiliations:** ^1^Laboratory of Ethnopharmacology, Institute of Integrated Traditional Chinese and Western Medicine, Xiangya Hospital, Central South University, Changsha, Hunan 410008, China; ^2^Medical Examination Center, The Third Xiangya Hospital, Central South University, Changsha, Hunan 410006, China; ^3^National Key Clinical Specialist Vocational School of TCM Encephalopathy, Xiangya Hospital, Central South University, Changsha, Hunan 410008, China; ^4^Institute of Diagnosis of Traditional Chinese Medicine, Hunan University of Traditional Chinese Medicine, Changsha, Hunan 410218, China

## Abstract

**Background:**

The traditional Chinese medicine formula Jiu Wei Zhen Xin Granula (JWZXG) is prescribed to treat generalized anxiety disorder (GAD) in China. This study was to assess the efficacy and safety of JWZXG in patients with GAD.

**Method:**

Data were pooled from 14 randomized controlled trials involving the assessment of mean changes of Hamilton Anxiety Rating Scale (HAMA) total scores, response rates, adverse event rates, quality, publication bias, and risk of bias.

**Results:**

Pooled analysis showed no significant difference in response rate (risk ratio 1.01, 95% CI [0.93–1.08]; *Z* test = 0.17, *P* = 0.86) and no significant difference between JWZXG group and azapirones group (RR 0.69, 95% CI [0.45, 1.06]; *Z* test = 1.69, *P* = 0.09) in rate of adverse events. Though no difference exists between JWZXG group and azapirones group in HAMA total score from baseline, JWZXG group was inferior to selective serotonin reuptake inhibitors (SSRIs) group (WMD −0.93, 95% CI [−1.64, −0.23]; *Z* test = 2.6, *P* = 0.009) which had more adverse events than JWZXG group (RR 0.64, 95% CI [0.46, 0.89]; *Z* test = 2.63, *P* = 0.009).

**Conclusions:**

This meta-analysis preliminarily suggests that JWZXG is as effective as azapirones, though having the same possibility of suffering AEs. JWZXG was inferior to SSRIs but causes fewer AEs in the treatment of GAD.

## 1. Introduction

Generalized anxiety disorder (GAD) is a prevalent and impairing disorder characterized by pervasive, excessive, and distressing worry [1]. Persons with GAD may be associated with muscle tension, somatic symptoms, and an exaggerated startle response. GAD has a 12-month prevalence of 3.1 percent in the United States [2] and of 1.0 percent in Europe [3]. Additionally, GAD is one of the most common anxiety disorders in the primary healthcare [4] and associated with a significant economic and social burden owing to reduced ability to work productively, and the degree of impairment is similar to that of major depression [5]. Sertraline, escitalopram, and paroxetine are the common used pharmaceuticals for GAD therapy [6]. However, while often effective, selective serotonin reuptake inhibitors (SSRIs) have efficacy limitations, such as failure to respond in many patients, delayed-onset of anxiolytic action, and risk of recurrence. Moreover, some patients taking SSRIs suffer obvious adverse events, such as suicidal ideation, sexual dysfunction, and dependency [7, 8]. Herbal medicine is increasing markedly in the treatment of mild to moderate mental disorders [9, 10], and growing evidences from systematic reviews and meta-analyses have confirmed the efficacy of some herbal preparations in the treatment of psychiatric disorders [11, 12]. Also, many clinic trials showed herbs likes Passion Flower [13], Kava [14], and chamomile [15, 16] and TCM prescriptions such as Gamisoyo-San [17] produced a clinically meaningful reduction in GAD symptoms. In China, Jiu Wei Zhen Xin Granula (JWZXG), developed from Ping Bu Zhen Xin Dan, has been prescribed to treat GAD, alone or in combination with other anxiolytics in recent years. JWZXG contains nine herbs: Panax Ginseng (ginseng), Spina Date Seed (seed of wild jujube),* Schisandra chinensis* (the fruit of Chinese magnolia-vine),* Poria cocos* (hoelen), Radix Polygalae (root of* Polygala tenuifolia* Willd), Rhizoma Corydalis (corydalis tuber), Radix Asparagi (Cochinchinese Asparagus Root),* Rehmannia glutinosa* (prepared* Rehmannia* root), and Cinnamon (cassia bark). Furthermore, ginsenosides [18], saponins, flavones, alkaloids [19, 20], dibenzocyclooctadiene lignans [21], polysaccharides [22], triterpenoid saponin [23], oligosaccharide ester [24], and 5-hydroxymethyl furfural [25] are the most active ingredients of these Chinese herbs mentioned above, and these ingredients are the markers for quality control.

Survey studies found that Spina Date Seed and* Poria cocos* are the most frequent traditional Chinese medicine in the treatment of anxiety disorder [26, 27]. Moreover, Panax Ginseng, Schisandra Chinensis, and Rhizoma Corydalis are commonly used as tranquillizing Chinese herbs [28]. Preclinical pharmacological research reveals potential anxiolytic-like mechanism of the active compounds from several individual herbs within JWZXG. For example, ginsenosides from Panax Ginseng exerts anxiolytic-like effects, in which the mechanism of action appears to be related to the GABAergic transmission [29]. Spinosin from Spina Date Seed is associated with the modulation by GABAA and 5-HT_1_A receptors [30, 31]. Lignans from* Schisandra chinensis* and 3,6′-disinapoyl sucrose from Radix Polygalae seem to play a significant role in modulating hyperactive HPA axis [32, 33]. Tetrahydropalmatine from Rhizoma Corydalis mediates anxiolytic activity through benzodiazepine site of GABAA receptor [34]. Additionally, flavones and saponins from Spina Date Seed and polygalasaponins from Radix Polygalae exert potential sedative-hypnotic activities [35, 36]. It is noteworthy that JWZXG acts through multitarget and multipathway; thus GAD with complex mechanisms is more likely to respond well to the treatment with JWZXG.

It has been only a few years since JWZXG has been used for the treatment of GAD; the efficacy and safety of utilizing JWZXG to treat GAD have just begun to be rigorously estimated in clinical studies, and evidences on the efficacy and safety of JWZXG have not been systematically assessed. Therefore, we aimed to assess the efficacy and safety of JWZXG compared to the conventional anxiolytics, such as buspirone, tandospirone, sertraline, and paroxetine, in the treatment of adult GAD.

## 2. Methods

### 2.1. Search Method for Inclusion of Studies

We systematically investigated the published reports on MEDLINE, PubMed, Cochrane Central Register of Controlled Trials (CENTRAL), Embase, CNKI, Wanfang Data, VIP Information, and Google Scholar to June 2017. We used the search terms “random”, “GAD”, “Generalized Anxiety Disorder”, “Generalized Anxiety Disorder”, “Jiu Wei Zhen Xin”, “Jiuweizhenxin”, and “JWZX” to identify that studies referred to randomized controlled trials (RCTs) involving JWZXG in the treatment of GAD.

### 2.2. Study Selection

Two investigators independently screened titles and abstracts to determine which trials were eligible for this meta-analysis. Discrepancies were resolved by discussing with a senior investigator.

Inclusion criteria were described as follows: (1) the experiments were conducted with randomized and controlled design; (2) GAD diagnosis should be accomplished based on International Classification of Diseases Tenth Revision (ICD-10), Diagnostic and Statistical Manual of Mental Disorders-4th Edition (DSM-IV), or Chinese Classification of Mental Disorders, Third Edition (CCMD-3); (3) the subjects should be adult patients; (4) the experiments should include the comparison of the efficacy of JWZXG and anxiolytics; (5) sample size should be more than 60; (6) outcome measures should include the clinical efficacy and rates of adverse events (AEs) during therapy.

The primary efficacy assessment was the mean change in Hamilton Anxiety Rating Scale (HAMA) total score from baseline to endpoint [37]. The secondary outcome was measured by response rates (≥50% decrease of baseline score in HAMA) [38].

In addition, the exclusion criteria include the following: (1) studies involved patients complicated with other mental disorders; (2) studies compared the efficacy of JWZXG to psychological therapy alone or compared the efficacy of JWZXG to JWZXG plus anxiolytics; (3) studies did not contain original data.

### 2.3. Data Extraction

Two reviewers independently extracted data, and the following data were extracted from eligible trials: (1) information of the publication (first author, year, and journal); (2) age and gender distribution of patients, number of patients in each arm, and severity and duration of the disease; (3) diagnostic criteria and outcome assessments; (4) dosage and treatment duration of intervention and control medicines; (5) methodological quality: evaluation of randomization, blinding, handling of attrition, and allocation concealment. When necessary, we contacted the authors to obtain missing information about trials.

### 2.4. Quality Appraisal

Methodological quality was evaluated primarily by Jadad's validated score, and allocation concealment was also assessed [39, 40]. Disagreements were resolved by discussing with a third reviewer.

### 2.5. Sensitivity Analysis

Sensitivity analyses were carried out to examine the robustness of the overall effect size. Each of the trials with poor methodological quality (Jadad score ≤ 2) or at high risk of bias was removed in turn from the analysis to investigate the changes of effect size and the influence on heterogeneity [41].

### 2.6. Publication Bias

Publication bias is a potential bias in systematic reviews and meta-analysis. An indication of publication bias is an asymmetrical funnel plot. However, other study factors, such as citation bias, true heterogeneity, intensity of intervention, data irregularity, and poor methodological design, also lead to asymmetry [42]. Therefore, the likelihood of publication bias in the meta-analysis was assessed by asymmetry funnel plot and examined by Egger's and Begg's test statistic [43, 44].

### 2.7. Assessment of Risk of Bias

**Figure 1:**
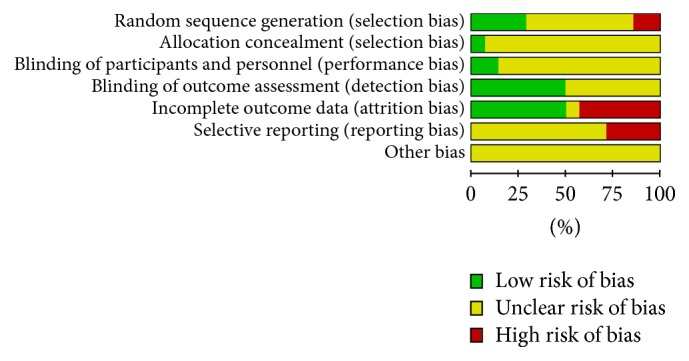
Risk of bias graph: authors' judgements about each risk of bias item presented as percentages across all included studies.

**Figure 2:**
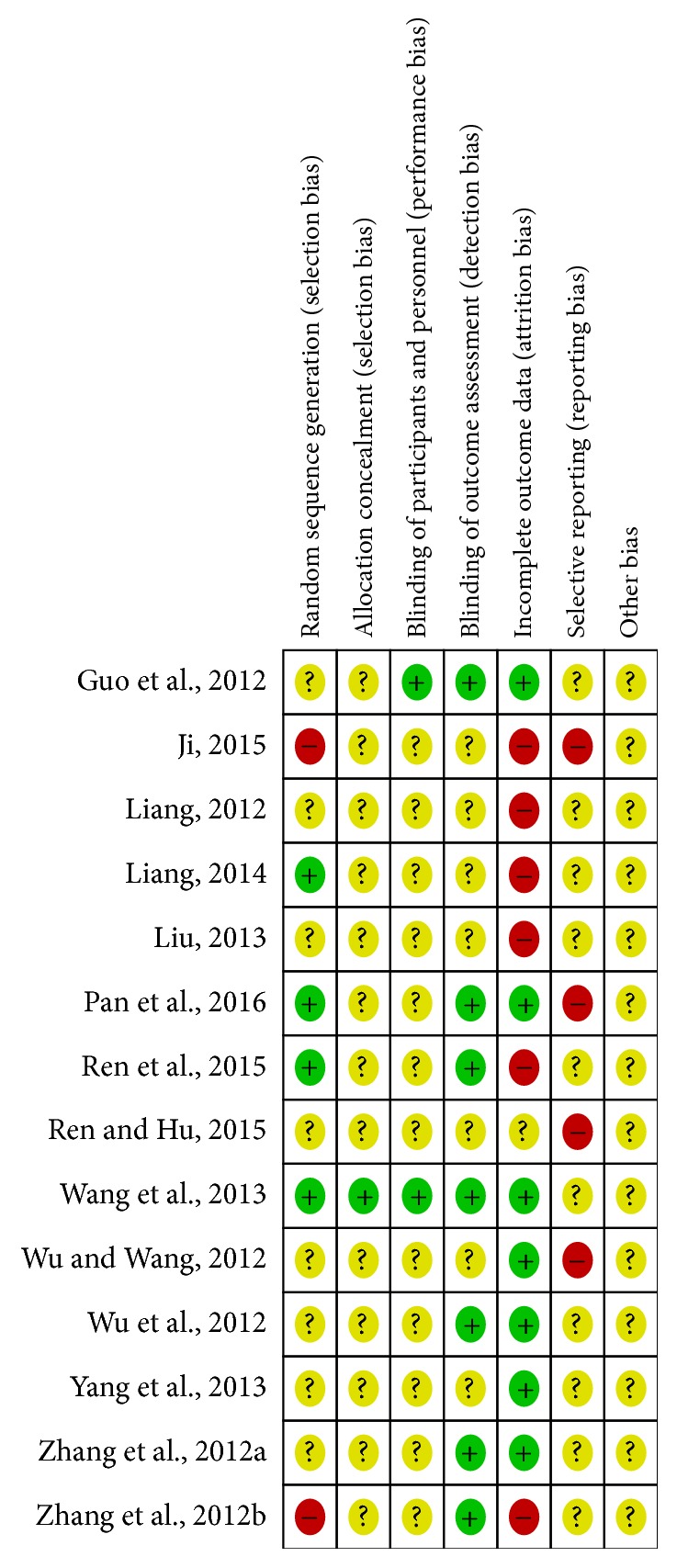
Risk of bias summary: authors' judgements about each risk of bias item for each included study.

The risk of bias was evaluated independently by two reviewers; and disagreements were resolved by discussing with a third assessor. We assessed the risk of bias using the seven factors set out from the Cochrane Handbook for Systematic Reviews of Interventions [45]. Study was rated as low risk of bias, high risk of bias, or unclear risk of bias using the Cochrane Risk of Bias tool, and the results were displayed in Figures 1 and 2. In general, the validity of this meta-analysis was regarded as high risk due to the relative lacking of specific information.

### 2.8. Statistical Analysis

We used inverse variance (IV) method to calculate weighted mean difference (WMD) and 95% confidence interval (95% CI) for continuous database. For dichotomous data, risk ratios (RRs) with 95% CI were calculated using Mantel-Haenszel (M-H) method. An alpha level of 0.05 was used for statistical significance.

Heterogeneity between trials was assessed using Cochran's *Q* statistic and Higgins' *I* squared statistic. The *Q* statistic is a weighted sum of squared deviations of individual study's effect estimate from the overall effect estimate. A *P* value for Chi-square less than or equal to 0.10 is considered to be of significant heterogeneity [46]. *I* squared statistic indicates the percentage of observed variation due to between-study heterogeneity rather than sampling error; a value of 0% indicates no significant heterogeneity, 25% means low heterogeneity, 50% means moderate heterogeneity, and 75% means high heterogeneity [47]. A fixed-effect model was applied when statistical homogeneity existed (*P* value > 0.1 or *I*2 < 50%) and a random-effect model was applied when statistical heterogeneity appeared (*P* value < 0.1 or *I*2 > 50%).

All analyses were calculated with Review Manager version 5.3 software (Cochrane Collaboration) and STATA software version 14.0 (STATA Corporation, College Station, TX, US).

## 3. Results

### 3.1. Studies Selection

**Figure 3:**
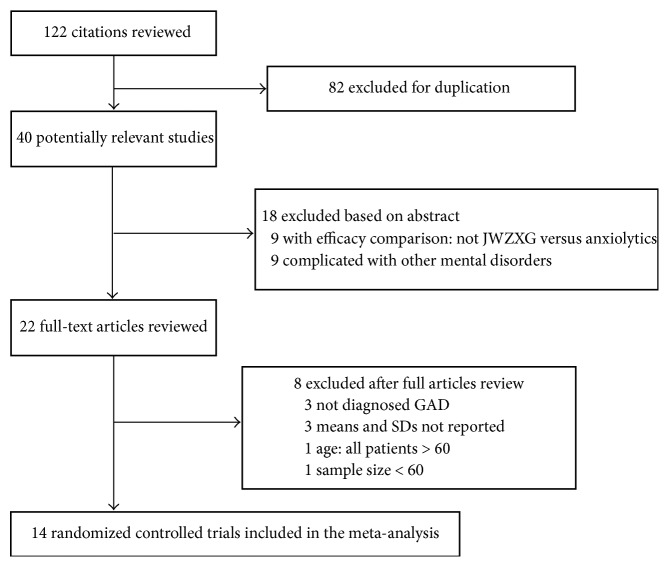
The study selection flowchart.

A total of 122 published trials involving JWZXG in the treatment of GAD were identified with the search strategy. Among 14 eligible studies 1358 participants were finally enrolled in the meta-analysis; all included trials were performed and reported in China. The study selection flowchart is presented in Figure 3.

The included trials were published from April 2012 to December 2016, and the sample size varied considerably from 60 to 448. 4 trials used buspirone in the control group [48–51]. Tandospirone [52–55], paroxetine [56–58], escitalopram [59], and sertraline [60, 61] were used as controls in other studies. Therapy duration ranged from 4 to 8 weeks, only 2 trials [50, 52] lasted 4 weeks, 9 trials [48, 49, 52, 55–60] lasted 6 weeks, and the remaining 3 trials [51, 54, 61] lasted 8 weeks. Details of included trials were summarized in Table 1.

### 3.2. Methodological Quality

Except that 1 trial [50] was of multicenter, randomized, and placebo-controlled design, the remaining 13 included trials that were single center, randomized, and controlled studies (Table 1). Four trials [50, 52, 57, 58] were reported using an adequate randomization method by means of random digit table. Two studies [48, 50] were reported using an adequate allocation concealment method. Two studies [48, 50] were double-blind trials and 1 trial [57] was single-blind (assessor-blind). Six studies [48, 49, 55–58] provided information on dropouts and the reasons. For the other sources of bias, 13 studies reported that there was no difference in baseline (e.g., age, sex, and course of disease).

Seven trials reached a Jadad score of 1, 2 trials reached a score of 2, 3 trials reached a score of 3, 1 trial reached a score of 4, and the remaining 1 trial reached a score of 5 (Table 1).

### 3.3. Comparison of the Mean Change in HAMA Total Score between JWZXG and Anxiolytics

**Figure 4:**
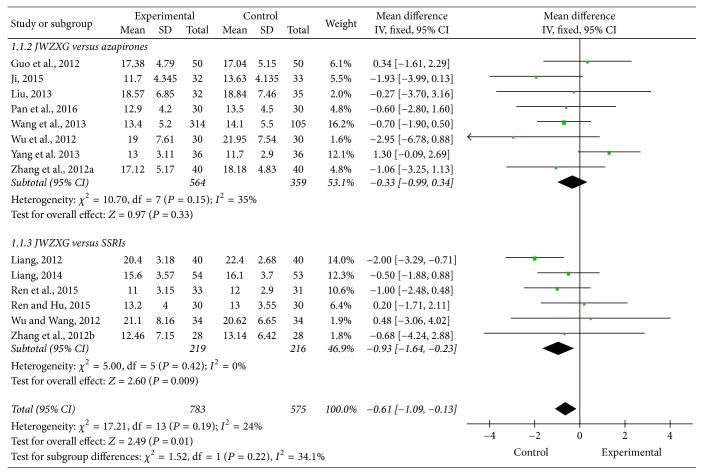
Comparison of the mean change in HAMA total score between JWZXG and anxiolytics under fixed-effects model.

**Figure 5:**
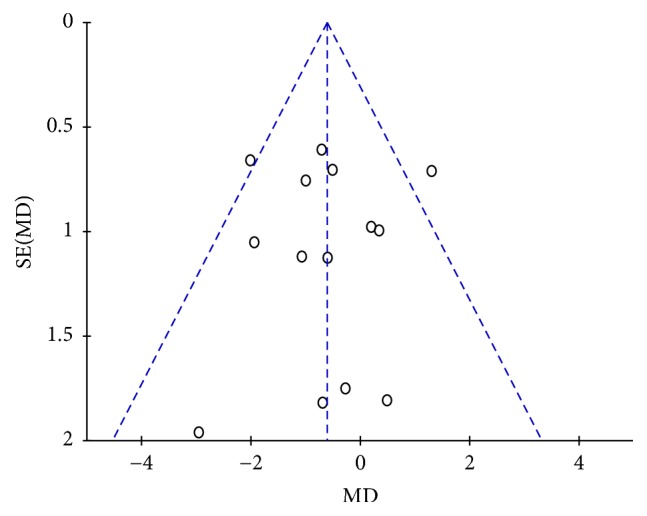
Funnel plot of comparison of the mean change in HAMA total score between JWZXG and anxiolytics.

All the included trials (*n* = 1358, 783 patients in the JWZXG treatment arms and 575 in the control arms) contributed to this analysis. As indicated in Figure 4, the pooled weight mean difference (WMD) was −0.61 (95% CI [−1.10, −0.13]; *Z* test = 2.49, *P* = 0.01) under the fixed-effects model, which suggested the control group is more effective than the experimental group in mean change of the HAMA total score from baseline. In the subgroup of azapirones and SSRIs, the pooled weight mean difference (WMD) was −0.33 (95% CI [−0.99, 0.34]; *Z* test = 0.97, *P* = 0.33) and −0.93 (95% CI [−1.64, −0.23]; *Z* test = 2.6, *P* = 0.009), respectively, showing no significant difference in mean change of the HAMA total score from baseline between JWZXG group and azapirones group, and the effect in mean change of the HAMA total score of SSRIs group was better than JWZXG group. There was moderate heterogeneity (*P* = 0.19, *I*2 = 24%). Funnel plot (Figure 5), Begg's test (*P* = 0.584), and Egger's test (*P* = 0.856) did not indicate the presence of publication bias. Sensitivity analysis showed 2 trials [54, 60] seemed to markedly influence the pooled WMD: a significant advantage of JWZXG compared to anxiolytics in terms of mean change in HAMA total score was found (WMD −0.39, 95% CI [−0.91, −0.13]; *Z* test = 1.46, *P* = 0.14), and heterogeneity was reduced to *P* = 0.45, *I*2 = 0%, when removing this trial [60] from the analysis; a significant advantage of JWZXG compared to anxiolytics in terms of mean change in HAMA total score was found (WMD −0.88, 95% CI [−1.39, −0.36]; *Z* test = 3.34, *P* = 0.0008), and heterogeneity was reduced to *P* = 0.71, *I*2 = 0%, when removing this trial [54] from the analysis.

### 3.4. Assessment of the Response Rate of JWZXG versus Anxiolytics

**Figure 6:**
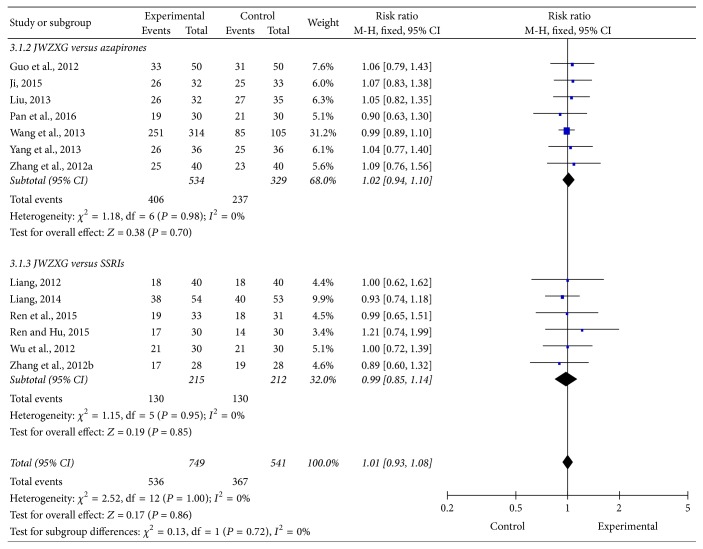
Comparison of the response rate between JWZXG arm and anxiolytics arm under fixed-effects model.

**Figure 7:**
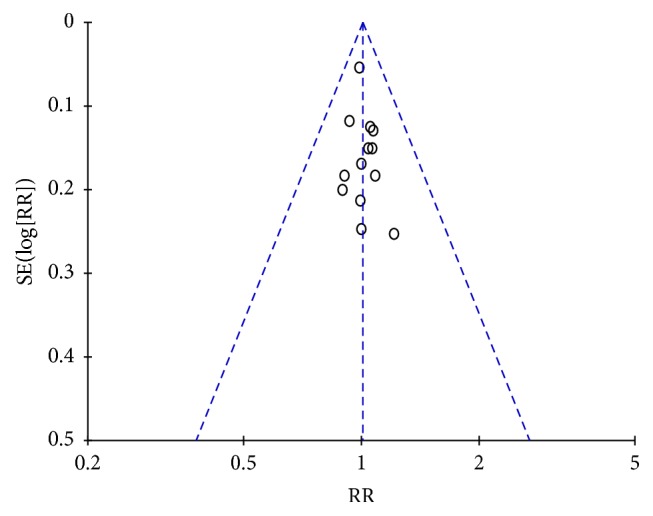
Funnel plot of comparison of the response rate between JWZXG arm and anxiolytics arm.

In all the included trials except one [60], JWZXG was comparable with anxiolytics in terms of response rate. As indicated in Figure 6, a meta-analysis of 13 trials (*n* = 1290, 749 patients in the JWZXG treatment arms and 541 in the control arms) showed no significant difference in response rates between JWZXG and control groups (RR 1.01, 95% CI [0.93–1.08]; *Z* test = 0.17, *P* = 0.86). In the subgroup, there was also no significant difference. Fixed-effects model was used according to the test of heterogeneity among the trials (*P* = 1.00, *I*2 = 0%). Visual inspection of funnel plot (Figure 7), Egger's test (*P* = 0.407), and Begg's test (*P* = 0.855) did not show the publication bias. A sensitivity analysis was performed to examine the robustness of the pooled RR for response rate, and no significant influence on the pooled RR for response rate was found.

### 3.5. Rates of Adverse Events (AEs)

**Figure 8:**
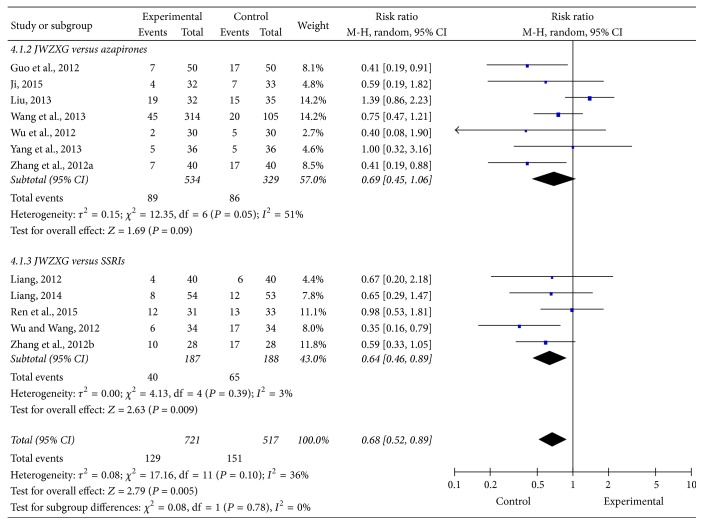
Comparison of AE rates between JWZXG and anxiolytics treatment.

**Figure 9:**
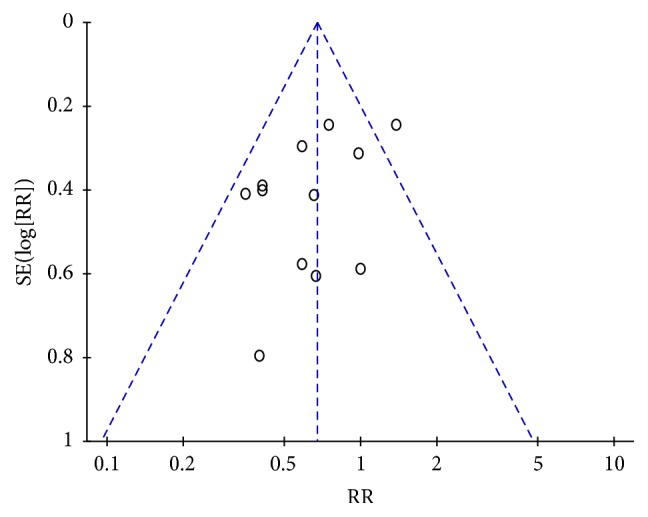
Funnel plot of comparison of AE rates between JWZXG and anxiolytics treatment.

All the included trials, except 2 trials [52, 59], reported rates of AEs. Five studies [49, 51, 55, 58, 60] found no significant differences in rates of AEs between JWZXG and anxiolytics, whereas 3 studies [50, 53, 56] suggested better tolerance of JWZXG than anxiolytics, and the differences were significant. The meta-analysis showed that patients in JWZXG were significantly less likely to suffer AE compared to anxiolytics (Figure 8). The pooled risk ratio (RR) for the rate of AE was 0.67 (95% CI [0.45, 0.83]; *Z* test = 2.79, *P* = 0.005). But in the subgroup the pooled risk ratio (RR) for the rate of AE of azapirones group and SSRIs group was 0.69 (95% CI [0.45, 1.06]; *Z* test = 1.69, *P* = 0.09) and 0.64 (95% CI [0.46, 0.89]; *Z* test = 2.63, *P* = 0.009), respectively, suggesting no significant difference between JWZXG group and azapirones group, and the JWZXG group was significantly less likely to suffer AE than SSRIs group. We used random-effects model according to the test of heterogeneity among the included trials (*P* = 0.10, *I*2 = 36%). Funnel plot (Figure 9), Egger's test (*P* = 0.162), and Begg's test (*P* = 0.436) did not show the publication bias. Sensitivity analysis showed 1 trial [49] seemed to markedly influence the pooled risk ratios. The risk ratio was 0.60 (95% CI [0.48, 0.76]; *Z* = 4.32, *P* < 0.00001), and heterogeneity was *P* = 0.64, *I*2 = 0%, when this trial was removed from the analysis.

## 4. Discussion

This meta-analysis identified 14 trials with a large number of participants (*n* = 1358) and examined the efficacy and safety of JWZXG in GAD. Pooled analysis showed there was a significant difference in terms of mean change of HAMA total score (WMD −0.61, 95% CI [−1.10, −0.13]; *Z* test = 2.49, *P* = 0.01) in total events, but in the subgroup though data showed SSRIs group has better effect on the mean change of HAMA total score (WMD −0.33, 95% CI [−0.99, 0.34]; *Z* test = 0.97, *P* = 0.33), no significant difference was between JWZXG group and azapirones group (WMD −0.93, 95% CI [−1.64, −0.23]; *Z* test = 2.6, *P* = 0.009), indicating that JWZXG was at least as effective as azapirones, and there was no significant difference in response rate (RR 1.01, 95% CI [0.93, −1.08]; *Z* test = 0.17, *P* = 0.86). However, JWZXG is better tolerated than SSRIs, causing fewer AEs (RR 0.64, 95% CI [0.46, 0.89]; *Z* test = 2.63, *P* = 0.009). Based on the results, it appeared that JWZXG was an effective preparation for treating GAD with lower risk of severe AEs than SSRIs. However, the results should be interpreted with more caution due to the methodological problems, short treatment duration, lack of placebo group, and small number of the included studies.

GAD is one of the most common anxiety disorders in adults and requires adequate long-term therapeutic management [8]. Herbal medicines, which could calm the mind and enhance positive mood, have been used for centuries, and the increasing numbers of patients with anxiety disorder have been treated with herbal medicines in the western world as well [62]. Although herbs and their preparations are proven to be effective in treating GAD [63], one of the biggest problems for the acceptance of the preparations is the lack of standardization of these preparations [64]. JWZXG is a Chinese patent medicine with a modern dosage for GAD, which ensures standardization of quality and properties of the individual Chinese herb, safety, and efficacy of the preparation to a certain extent [65]. Moreover, JWZXG is used with the same dosage and usage (6 g/d, tid) in the included trials, which probably enhances clinical homogeneity of the included trials. In addition, JWZXG is a granula preparation and it is possible to prepare herbal formulas and placebo in granula to achieve the placebo design in RCTs.

The meta-analysis showed that the rate of AEs in JWZXG group was significantly lower than SSRIs group. The common side effects of JWZXG included dry mouth, constipation, dizziness, and nausea [50]. Treatment duration lasted 4–8 weeks in the included trials; therefore, the long-term safety of JWZXG was not considered. The side effects of overdose were also not reported and when JWZXG has nonresponse whether and how the patients increase the dose and when the patients have severe AEs how to reduce the dose need to be further investigated. Moreover, it cannot exclude lack of Nocebo effect in placebo group in trials that are included in this study. Furthermore, herb-drug interaction is an important safety issue [66]. For instance, the primary Chinese herb, Panax Ginseng, in JWZXG has been reported to interact with warfarin, phenelzine, and alcohol [67]. Thus, further studies are needed to determine the potential interactions between JWZXG and synthetic drug.

Statistical and methodological problems of the included studies limited the external validity of the results. For example, approaches of randomization and allocation concealment, which could impact selection biases and exaggerate the estimates of effect [40], were not described clearly in most of the included trials, as well as nonblinding or inadequate blinding which could cause selection and measurement bias and also overstate the estimates of treatment effects and AEs [68]. Although we searched the literature with no restriction to language, all the studies included in the meta-analysis were performed and published in China, so publication bias exists. Hence, further studies with excellent methodological quality and long-term efficacy assessment are definitely required to exactly determine the exact efficacy and safety of JWZXG for GAD.

## 5. Conclusions

In summary, our meta-analysis preliminarily suggests that JWZXG is as effective as azapirones, though the same possibility of suffering AEs exists. JWZXG was inferior to SSRIs but causes fewer AEs in the treatment of GAD. However, the methodological limitation, short treatment duration, and small number of the included studies may limit the external validity of the results. Further studies with excellent methodological quality and long-term efficacy assessment are needed to further determine the exact efficacy and safety of JWZXG for GAD.

## Figures and Tables

**Table 1:** Details of the included trials for JWZXG in the treatment of GAD.

Study	Men/total	Age	Diagnostic criteria	Therapy duration	Interventions		Response definition	Method	Jadad scores	Dropout rate
					T	C				
Guo et al., 2012	T 29/50 C 26/50	T 40.8 ± 13.2 C 43.2 ± 14.3	CCMD-3 HAMA ≥ 14	6 w	JWZX 18 g/d	Buspirone 15–60 mg/d	HAMA, TESS, response rate	Randomization, blinding of experimenter, participants, and assessors (placebo)	3	NR

Liang, 2012	T 18/40 C 19/40	T 40.5 ± 11.4 C 41.0 ± 10.9	CCMD-3 HAMA > 14	6 w	JWZX 18 g/d	Sertraline 50–100 mg/d	HAMA, TESS, response rate	Randomization	1	NR

Liang, 2014	T 22/54 C 20/53	T 36.5 ± 4.3 C 35.6 ± 4.2	CCMD-3 HAMA ≥ 14	6 w	JWZX 18 g/d	Paroxetine 10–40 mg/d	HAMA, TESS, response rate	Randomization (random digit table), dropouts	3	T 2 C 3

Liu, 2013	T 13/32 C 15/35	T 37.7 ± 8.6 C 38.7 ± 9.1	ICD-10 HAMA ≥ 14	6 w	JWZX 18 g/d	Buspirone 15–30 mg/d	HAMA, TESS, response rate	Randomization, dropouts	2	T 5 C 6

Wang et al., 2013	T 140/336 C 41/111	T 42 ± 14 C 43 ± 13	CCMD-3 HAMA ≥ 14	4 w	JWZX 24.1 ± 4.0 g/d	Buspirone 24.5 ± 4.3 mg/d	HAMA, TESS, response rate	Randomization (random digit table), dropouts, blinding of experimenter, participants, and assessors (placebo)	5	T 22/337 C 6/111

Wu et al., 2012	T 12/30 C 14/30	T 31.5 ± 15.5 C 33.5 ± 13.2	CCMD-3 HAMA ≥ 14 SAS ≥ 50	6 w	JWZX 18 g/d	Tandospirone 30 mg/d	HAMA, SAS, TESS response rate	Randomization, blinding of assessors	1	NR

Wu and Wang, 2012	32/68	34.4 ± 4.9	ICD-10 HAMA ≥ 14 SAS ≥ 50	8 w	JWZX 18 g/d	Sertraline 50 mg/d	HAMA, TESS	Randomization	1	NR

Yang et al., 2013	T 14/36 C 13/36	T 33.5 ± 11.8 C 32.5 ± 12.1	CCMD-3 HAMA ≥ 14	8 w	JWZX 18 g/d	Tandospirone 15–60 mg/d	HAMA, TESS, response rate	Randomization	1	NR

Zhang et al., 2012a	T 22/40 C 21/40	T 42.2 ± 15.5 C 42.7 ± 15.0	CCMD-3	6 w	JWZX 18 g/d	Buspirone 15–60 mg/d	HAMA, TESS, response rate	Randomization, blinding of assessors	1	NR

Zhang et al., 2012b	T 14/30 C 15/30	T 38.4 ± 13.7 C 38.9 ± 12.9	CCMD-3 HAMA ≥ 14	6 w	JWZX 18 g/d	Paroxetine 20 mg/d	HAMA, CGI, response rate	Randomization, dropouts, blinding of assessors	2	T 2 C 2

Pan et al., 2016	T 12/30 C 15/30	T 36 ± 7.1 C 39.1 ± 9.6	CCMD-3	4 W	JWZX 18 g/d	Tandospirone 15–30 mg/d	HAMA, response rate	Randomization, blinding of assessors	3	NR

Ren et al., 2015	T 12/36 C 11/36	T 34.6 ± 15.2 C 35.2 ± 13.9	CCMD-3	6 W	JWZX 18 g/d	Paroxetine 10–40 mg/d	HAMA, TESS, response rate	Randomization, dropouts	4	T 3/36 C 4/36

Ren and Hu, 2015	NR	T 43.2 ± 6.7 C 42.4 ± 9.3	CCMD-3	6 W	JWZX 18 g/d	Escitalopram 5–15 mg/d	HAMA, response rate	Randomization	1	NR

Ji, 2015	T 19/32 C 18/33	T 36.2 ± 11.4 C 38.5 ± 12.8	CCMD-3	6 W	JWZX 18 g/d	Tandospirone 15–60 mg/d	HAMA, TESS, response rate	Randomization, dropouts	1	T 2 C 3

GAD: generalized anxiety disorder; JWZXG: Jiu Wei Zhen Xin Granula; CCMD-3: Chinese Classification of Mental Disorders, Third Edition; ICD-10: International Classification of Diseases Tenth Revision; HAMA: Hamilton Anxiety Rating Scale; SAS: Self-Rating Anxiety Scale; TESS: Treatment Emergent Symptom Scale; NR: not reported; T: treatment group; C: control group.
